# Brain metastasis in colorectal cancer presenting as refractory hypertension

**DOI:** 10.1080/20009666.2018.1490138

**Published:** 2018-08-23

**Authors:** Syed Moin Hassan, Ateeq Mubarik, Salman Muddassir, Furqan Haq

**Affiliations:** a Internal Medicine Resident, Oak Hill Hospital, Brooksville, FL, USA; b Hospital Corporation America (HCA), West Florida Division, Tampa, FL, USA

**Keywords:** Brain metastasis, colorectal cancer, hypertensive urgency, neurosurgery, whole-brain radiotherapy, stereotactic radiosurgery, solitary lesion, mass effect

## Abstract

**Background**: Brain metastasis (BM) from colorectal cancer (CRC) is rare with the incidence ranging from 0.6% to 3.2%. There is also an increased incidence of BM with rectal primaries and is consistent with this patient’s presentation. Overall, there is scarce literature on the symptoms of patients who present with CRC BMs.

**Objectives**: We present a case of brain metastasis in colorectal cancer presenting with hypertensive urgency and severe headache.

**Methods and results**: This case highlights that neurological deficits are not necessary for BMs in patients with CRC and summarizes and reviews the associated literature regarding BM in CRC. A 57-year-old female with a past medical history of recently diagnosed stage IV moderately differentiated distal rectal adenocarcinoma with liver and lung metastasis was admitted with the primary complaint of hypertensive urgency, severe headache, intractable nausea and vomiting, and diarrhea. Magnetic resonance imaging brain showed a left cerebellar lesion measuring 3.6 × 3.2 × 2.9 cm, ipsilateral transtentorial herniation, and obliteration of the fourth ventricle. The patient was started on steroids and transferred for an urgent neurosurgical intervention to a tertiary care center.

**Conclusions**: Even though BMs are rare in CRC, clinicians should have a high index of suspicion with complaints like hypertensive urgency, headache, nausea, vomiting, vertigo, and blurring of vision triggering imaging studies to rule out BM. The approach to BM has become increasingly individualized as surgical and radiosurgical therapies have continued to evolve

**Abbreviations**: CRC: Colorectal cancer; BM: Brain metastasis; FOLFOX: Folinic acid, fluorouracil and oxaliplatin; CT: Computed tomography; IV: Intravenous; PO: By mouth; BAER: Brain auditory evoked response hearing testing; SSEP’s: Somatosensory evoked potentials; BMFI: Brain metastasis free interval; WBRT: Whole-brain radiation therapy; SRS: Stereotactic radiosurgery.

## 1. Background

Colorectal cancer (CRC) is the third most common cancer in men and women. It is estimated that 8.0% of all new cancer cases in the USA are CRC. It is also the second leading cause of mortality due to cancer in the USA [1]. There is scarce literature on the symptoms of patients who present with brain metastases (BMs). Our patient presented with hypertensive urgency associated with a headache which was refractory to treatment and without any focal neurological deficits. This case highlights that neurological deficits are not necessary for BMs in patients with CRC and summarizes and reviews the associated literature regarding BM in CRC. Informed consent from patient has been taken for publication.

## 2. Case presentation

**Figure 1.**
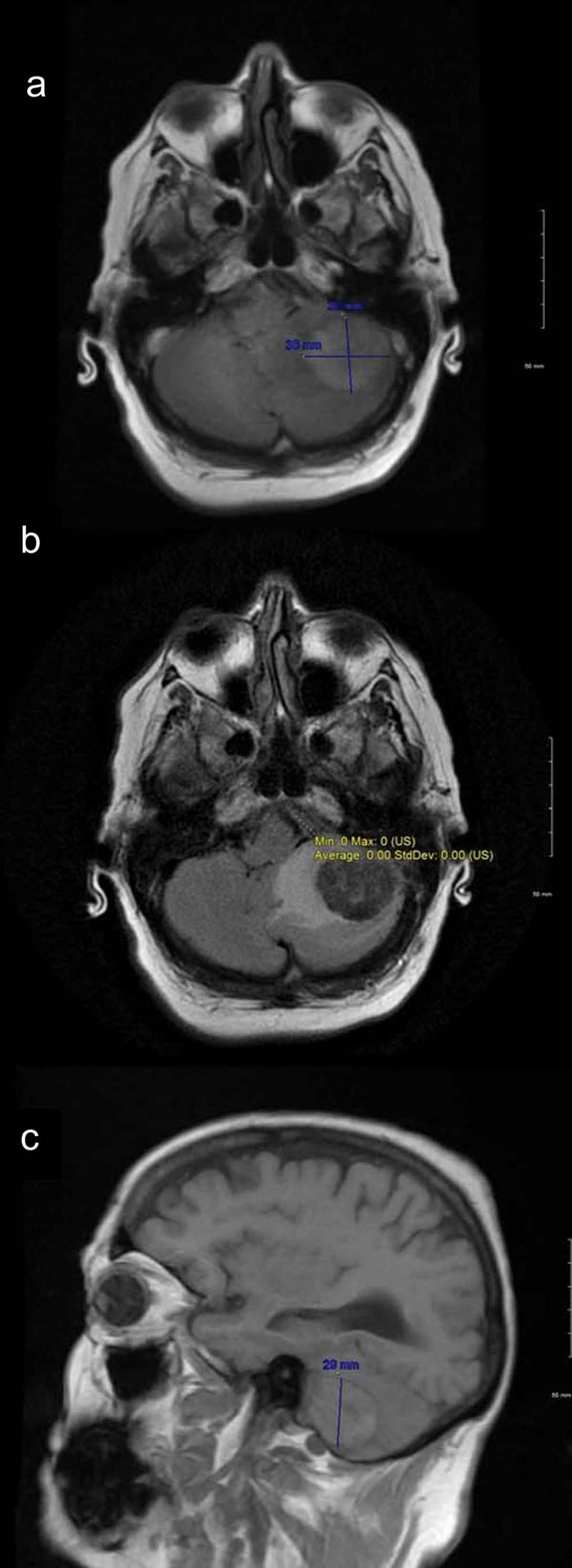
MRI brain showing left cerebellar lesion with significant amount of surrounding vasogenic edema, ipsilateral transtentorial herniation, and obliteration of the fourth ventricle.

**Figure 2.**
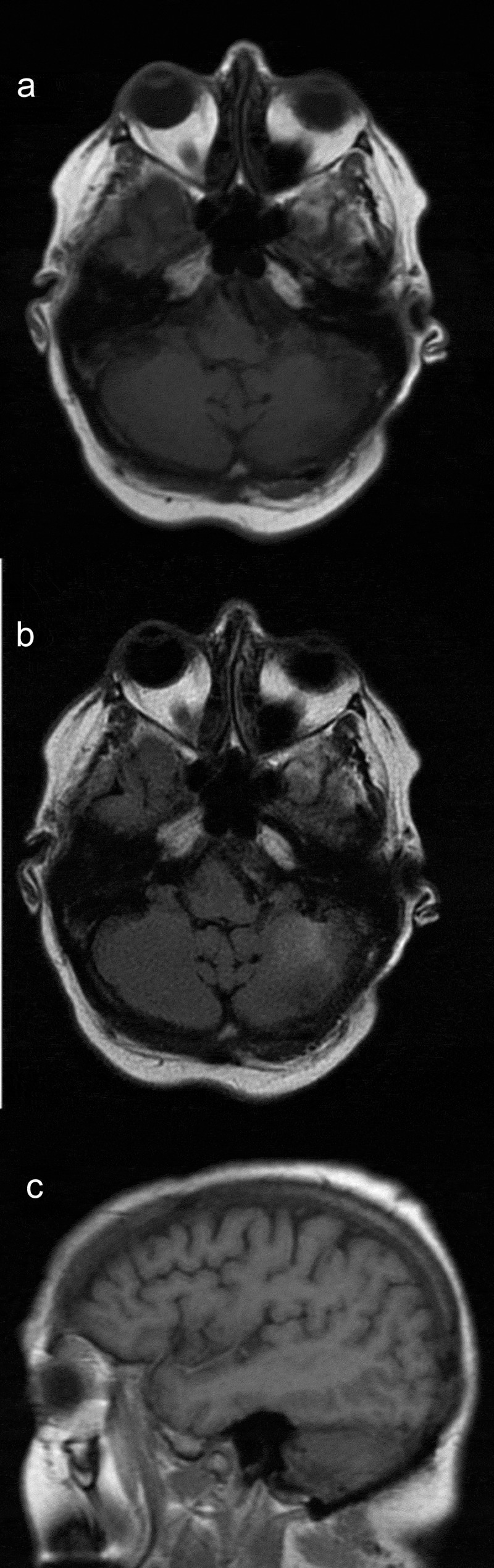
Follow-up MRI brain at 1 year showing evidence of prior resection of the left cerebellar mass with no recurrence of metastatic lesion or new metastasis.

Fifty-seven-year-old Caucasian female with a past medical history significant for hypertension and recently diagnosed stage IV moderately differentiated distal rectal adenocarcinoma with liver and lung metastasis status post second cycle of FOLFOX palliative chemotherapy 1 week ago was admitted with the primary complaint of hypertensive urgency with a severe headache, intractable nausea and vomiting, and diarrhea. At presentation, her blood pressure was 191/68. Examination did not show any focal neurological deficits. She was alert, awake, and oriented to time, place, and person; cranial nerves II–XII were intact; muscle power was five out of five bilaterally in upper and lower extremities; coordination was intact bilaterally; reflexes were 2+ bilaterally in upper and lower extremities; sensation was intact; and gait was normal. The case was discussed with oncologists who were of the view that symptoms may be due to hypertensive urgency versus BM (which are quite rare for CRC) or possible opiate withdrawal as the patient has been on high-dose opiates for her cancer-related pain. Computed tomography (CT) scan of the abdomen-pelvis did not show any evidence of bowel obstruction. Imaging of the brain would be considered if the patient did not improve with medical therapy. The patient was started initially on IV hydralazine but over the next 8 h patient blood pressure remained uncontrolled despite successive antihypertensives (IV labetalol, PO amlodipine, PO clonidine, IV enalaprilat, transdermal clonidine, IV metoprolol, and eventually IV nicardipine drip), ranging from 185/98 to 230/111. Brain imaging was ordered due to continuous severe headache and refractory hypertension. CT scan of the brain without contrast showed 3.3 × 2.3 × 2.8 cm hyperdense rounded mass in the region of the left cerebellum with surrounding vasogenic edema and a 5–6-mm shift of the posterior midline toward the right. Brain magnetic resonance imaging (MRI) with and without contrast showed left cerebellar lesion measuring 3.6 × 3.2 × 2.9 cm with high T1 signal intensity, low T2 signal intensity, and low gradient echo sequence signal intensity with peripheral and mild internal enhancement, and a significant amount of surrounding vasogenic edema, ipsilateral transtentorial herniation, and obliteration of the fourth ventricle (Figure 1). After discussion with the patient, the decision was made for urgent neurosurgical resection of the mass and subsequent whole-brain radiation therapy (WBRT). This decision was made as this was a solitary lesion which was symptomatic with a high degree of mass effect and edema in a patient with a good baseline functional status. The patient was started on IV dexamethasone 6 mg every 6 h, and transferred for an urgent neurosurgical intervention at a tertiary care center. The tumor was resected using an operative microscope, and a combination of suction and cautery. Postoperatively patient recovered well from the surgery and was discharged. Patient followed up outpatient and had WBRT in 4 weeks post discharge. On her 1-year follow-up, patient reported no new neurological deficits, and her repeat MRI brain (Figure 2) did not show any recurrence of her metastatic lesion or new metastasis.

## 3. Discussion

BM from CRC is rare with the incidence ranging from 0.6% to 3.2%. BM usually develops later in the course of disease, and patients often have concurrent metastasis to other organs when brain metastatic lesions are identified [2–4].

The duration from primary CRC diagnosis to BM diagnosis is reported between 20 and 40 months, and a shorter duration of 9–23 months is reported after metastatic CRC diagnosis to BM.

A trend of a shorter duration and increased incidence of BM is also seen with rectal primaries. The most common hypothesis explaining this trend emphasizes on the vascular anatomy with three main pathways: (1) portal vein to the liver, and from there to the lung, and thereafter brain. (2) Through the vena cava directly to the lung and thereafter the brain. (3). Through the vertebral plexus directly to the brain. This explains the decreased brain metastasis free interval and increased incidence of BM in lung metastases as compared to liver metastases and also the increased risk of BM in a rectal primary CRC. The increased risk due to a rectal primary CRC is also explained by the fact that the rectum drains more often through the vena cava vein rather than the colon. Our patient with a rectal primary, and lung and liver metastasis presentation is consistent with the above hypothesis, with a significantly deceased interval of 6 months between the diagnosis of primary CRC to the diagnosis of BM [4,5].

BMs have highly variable clinical features. Headache is the most common symptom present in patients with BM. In a study with 111 patients with CT or MRI identified primary or metastatic brain tumors, 48% of the patients had headache. In addition to headaches, nausea or vomiting was present in 40% of the patients. Majority of the patients had tension-type headache (77%). Early morning headache ‘classically’ associated with brain tumors was uncommon [6]. Other common symptoms included focal neurologic dysfunction which was present in 20–40% of patients, cognitive dysfunction, comprising of but not limited to memory problems and mood or personality changes, was present in 30–35% of patients [7], new onset of seizure was present in 10–20% of patients [8], and stroke was present in 5–10% of the patients [9]. However, there is scarce literature on the symptoms of patients who present with BMs in CRC. Damiens et al. describes presenting complaints of five patients who presented with headache and vertigo, blurring of vision, headache, vertigo and headache, and left hemiparesis, respectively [10]. In a recent study, Kim D-Y et al. looked at 19 patients with BM in CRC. The common presenting symptoms were headache in 42.1% and ataxia in 31.6% of the patients [11]. Our patient presented with hypertensive urgency with headache which was refractory to treatment and with no focal neurological deficits. Although headache is a common presentation in patients with BM in CRC, the combination with hypertensive urgency is a unique presentation.

The approach to BM has become increasingly individualized as surgical and radiosurgical therapies have continued to evolve. Factors influencing treatment strategy include type of cancer, metastasis size, metastasis site, solitary metastasis versus multiple metastases, functional status of patient, presence or absence of symptoms, degree of mass effect and edema, extent of systemic disease, and patient preference regarding invasive therapy [12,13]. Single, surgically accessible metastasis which is large and/or associated with significant edema and mass effect, like in our patient, should undergo surgical resection as it achieves rapid symptom relief and local control. Three randomized clinical trials have compared surgery plus WBRT with WBRT alone in patients with single BMs. The first trial included 48 patients with a single BM who were either treated with surgical resection followed by WBRT or WBRT alone. Patients with surgery followed by WBRT had significantly fewer local recurrences (20% versus 52%), improved survival (40 versus 15 weeks), and a better quality of life [14]. The second trial including 63 patients with a single BM showed improved overall survival with surgery and WBRT as compared to WBRT alone (10 versus 6 months). The subset of patients with stable extracranial disease was the one that benefited most from surgery (median survival 12 months), while patients with active extracranial disease did not appear to significantly benefit from surgery (median survival 5 months) [15]. The third trial had a subset of 43 patients that received radiation alone and 41 patients who received surgery plus radiation. Although no difference in survival was detected between the groups, a survival benefit in favorable prognosis patients might have been missed as a higher proportion of patients had a lower Karnofsky Performance Score at baseline (The Karnofsky Performance Score is an assessment tool for functional impairment with lower scores associated with decreased likelihood of survival) and extracranial disease [16,17].

WBRT has been the standard of care for BMs [18]. However with improving targeting technology, stereotactic radiosurgery (SRS) is emerging as an alternative method to treat metastatic disease to the brain. SRS has the advantage of requiring decreased time, increased efficacy against radioresistant tumors, decreased hair loss, and fewer neurocognitive side effects but has the disadvantage of increased likelihood of re-irradiation [19–22]. Currently in the age of new systemic therapies, the role of WBRT and SRS in the treatment of BMs is evolving [23]. Two studies, Aoyama et al. and Sneed et al., showed that there was no difference in survival between WBRT plus SRS and SRS alone. However Aoyama et al. did report an increased incidence of intracranial relapse in patients who received SRS alone [24,25]. A study by Chang et al. showed an improved 4-month overall survival in the SRS group and a higher decline in learning and memory in patients treated with both SRS and WBRT, but the local and distant control was better in the SRS plus WBRT group [19]. Nonetheless, it is important to note that Aoyama et al., Sneed et al., and Chang et al. looked at BM in general and not BMs from gastrointestinal (GI) primaries. A recent study, Sanghvi et al., found that survival and intracranial disease control were poor following RT for BMs from GI primaries. The outcomes were worse than published series for other primary malignancies metastatic to the brain. The authors postulated that the poor overall survival and local control could possibly be explained by the fact that BMs are a late event in most GI malignancies and present as diffuse active extracranial disease, and the radioresistant biology of GI malignancies. Sanghvi et al. further reported there was no significant difference in survival between patients who received SRS versus those who received WBRT and higher distant brain failure rates with SRS alone [26]. These studies highlight the fact that the choice of radiotherapy in BM should be made on a case-by-case basis. For our oncologist, after discussion with the patient, the decreased rate of intracranial relapse with the WBRT as compared to SRS outweighed the favorable side effect profile of SRS. This case highlights the fact that neurological deficits are not a necessary presentation for BMs in patients with CRC especially rectal primaries. Even though BM are rare in CRC, clinicians should have a high index of suspicion with complaints like hypertensive urgency, headache, nausea, vomiting, vertigo, and blurring of vision triggering imaging studies to rule out BM.
